# Supplementary material for: Asynchronous magnetic resonance elastography: Shear wave speed reconstruction using noise correlation of incoherent waves
Source: Magn Reson Med. 2022 Oct 27;89(3):990–1001. doi: 10.1002/mrm.29502 (PMC9792433; doi:10.1002/mrm.29502)
Supplement: Supplementary file 1 — DATA S1 MATLAB code used to generate the numerical simulations of Figure 1 [file MRM-89-990-s002.zip › k-Wave/helpfiles/example_ivp_sensor_frequency_response.html]

Defining A Gaussian Sensor Frequency Response Example (k-Wave)


# Defining A Gaussian Sensor Frequency Response Example

This example shows how to account for the frequency response of detectors when this response has a Gaussian shape (for example, piezoelectric ultrasound transducers). It builds on the Homogeneous Propagation Medium Example.

- Open the file in the MATLAB Editor
- Run the file in MATLAB

## Contents

- Defining the sensor frequency response
- Running the simulation

## Defining the sensor frequency response

The frequency response of the sensor is assigned using the `frequency_response` field of the `sensor` input structure. This accepts two parameters, one for the center frequency, and one for the bandwidth of the frequency response. The transducer bandwidth, defined as a percentage, controls the full-width at half-maximum (FWHM) of the filter, where the FWHM = % bandwidth \* center frequency.

```
% define the frequency response of the sensor elements
center_freq = 3e6;      % [Hz]
bandwidth = 80;         % [%]
sensor.frequency_response = [center_freq, bandwidth];
```

When an input for `sensor.frequency_response` is defined, a Gaussian filter is applied within the simulation functions after the simulation is completed by multiplying the Fourier transformed signals by a zero-phase Gaussian window using `gaussianFilter`. Note, the same filter can also easily be applied by calling `gaussianFilter` explicitly when the sensor data is returned.


## Running the simulation

To illustrate the effect of the filter, the time series recorded at the first sensor position for a simulation with and without the `sensor.frequency_response` field defined are plotted below. The corresponding amplitude spectrums are also shown. The removal of the low frequency information has a significant effect on the signal amplitude.
